# MMDet-Edge: A Multi-Scale and Multi-Object Detection Framework for Safety-Critical Edge Deployment

**DOI:** 10.3390/s26041151

**Published:** 2026-02-10

**Authors:** Tianyi Zhu, Hong Liu, Haoming Duan, Yiyang Liu, Jinjun Rao

**Affiliations:** 1 Shanghai Dahua Surveying and Mapping Technology Co., Ltd., Shanghai 201208, China; z15295370172@163.com (T.Z.); liuhong@cccc-sdc.com (H.L.); yephm996@163.com (H.D.); 2 Department of Precision Mechanical Engineering, School of Mechatronic Engineering and Automation, Shanghai University, Shanghai 200444, China; yoyogreen@shu.edu.cn

**Keywords:** edge computing, multi-class object detection, construction safety, adaptive feature fusion, neural architecture search, risk-aware optimization

## Abstract

Construction site safety remains a critical global challenge, demanding urgent attention. Existing surveillance systems struggle to balance multi-object detection accuracy, real-time efficiency, and environmental robustness under strict edge constraints. This paper presents MMDet-Edge, an edge-optimized unified detection framework that addresses these competing demands via three synergistic innovations. First, an adaptive feature fusion architecture with a learnable spatial–channel attention mechanism resolves cross-scale conflicts, boosting small-object average precision (AP) by 9.3%. Second, a hardware-conscious neural architecture search (HC-NAS) strategy co-optimizes sparsity patterns and quantization sensitivity, achieving a state-of-the-art performance of 89.4% mAP@0.5 at only 1.8 W power consumption—surpassing contemporary edge detectors by 6.3% mAP under equivalent power budgets. Third, by incorporating OSHA fatality statistics into a novel risk-weighted evaluation paradigm, we reduce high-consequence false negatives by 34%. Comprehensive evaluations on a purpose-built benchmark and cross-dataset tests demonstrate MMDet-Edge’s superiority. It outperforms a wide range of state-of-the-art models. Validated across three active construction sites, the system enables real-time detection of five safety-critical targets (personnel, helmets, flames, smoke, vests) under extreme conditions, including >60% occlusion and >100 lux illumination variance. Our field deployments demonstrated a 22% reduction in safety incidents compared to conventional systems, establishing a new architectural paradigm for safety-critical edge AI through principled hardware–algorithm co-design.

## 1. Introduction

### 1.1. Research Background and Significance

The rapid advancement of edge computing enables complex deep learning models to be deployed on resource-constrained devices, serving as a critical enabler for Industrial Internet of Things (IIoT) and smart city applications [1]. This is particularly paramount in safety-critical environments like construction sites, mines, and chemical plants, where real-time and reliable multi-object visual perception systems are essential. According to the International Labour Organization (ILO), the construction sector accounts for over 20% of occupational fatalities globally, with direct costs exceeding $170 billion annually. This underscores the limitations of traditional safety supervision methods [2].

Although deep learning-based object detection has achieved remarkable success in general domains [3,4], its deployment in safety-critical edge environments faces fundamental conflicts. First, there is an inherent trade-off between model accuracy and computational efficiency. High-precision detectors (e.g., two-stage or large single-stage models) typically involve high computational complexity, making it difficult to meet real-time requirements on power-stringent edge devices [5]. Second, the problems of scale variance and semantic interference in multi-class detection are drastically amplified in complex industrial settings. For instance, the feature representation requirements for small objects (e.g., safety helmets) and large objects (e.g., excavators) are inherently conflicting, while visually disparate categories (e.g., flames vs. reflective vests) interfere in the feature space, leading to significant performance degradation in general-purpose detectors [6]. Furthermore, edge-oriented model compression techniques (e.g., pruning and quantization), while improving efficiency, often introduce non-negligible accuracy loss, particularly degrading detection performance for small and heavily occluded objects [7].

Consequently, developing a unified framework that can co-optimize detection accuracy, inference efficiency, and environmental robustness is not only an urgent engineering need for building intelligent site monitoring systems but also a core academic challenge in advancing safety-critical edge artificial intelligence.

### 1.2. Current State of Research

Current research on vision-based construction safety monitoring primarily evolves along three trajectories:

#### 1.2.1. Specialized Single-Target Detectors

Early research focused on specific hazards, such as safety helmets [8], flames [9], and reflective vests [10]. These methods, leveraging models finely tuned for specific tasks, can achieve high accuracy under constrained conditions. However, comprehensive safety monitoring requires deploying multiple independent models, leading to system redundancy, multiplied resource consumption, and aggregate power consumption often exceeding 40 W, which is infeasible for solar-powered edge nodes [11]. This isolated model approach contradicts the efficiency principles of edge computing.

#### 1.2.2. Adoption of General-Purpose Detectors

To address the redundancy of multiple models, researchers have employed general detection frameworks like YOLO [12] and DETR [13] for unified multi-object detection. However, these models, designed for natural images, underperform in complex construction scenarios. Studies show that their Average Precision (AP) drops by over 30% for objects occupying less than 0.1% of the image area [6]. Moreover, beyond scale issues, semantic interference between categories (e.g., fire and personal protective equipment) further degrades performance [6]. The root cause is that general frameworks’ feature extraction and fusion mechanisms are not optimized for the extreme scale imbalance and large inter-class variance in industrial scenes.

#### 1.2.3. Exploration of Model Lightweighting Techniques

To adapt models to edge devices, numerous lightweight techniques have been proposed, including pruning [14], quantization [15], and Neural Architecture Search (NAS) [16,17]. However, most existing methods are designed for general tasks, exhibiting limitations in safety-critical contexts. For instance, aggressive channel pruning may inadvertently discard features crucial for discriminating small objects, while low-precision quantization can amplify localization errors and degrade the detection rate of occluded objects [7,15]. Moreover, most Hardware-Aware NAS (HW-NAS) studies [17] optimize for latency or energy consumption as single objectives, lacking co-optimization across accuracy, efficiency, and risk-aware capability.

In summary, existing research has yet to systematically resolve the accuracy–efficiency–robustness trilemma, lacking an end-to-end co-design perspective from algorithmic innovation to hardware deployment.

### 1.3. Main Contributions

To address the above challenges, this paper proposes MMDet-Edge, a multi-scale and multi-object detection framework for safety-critical edge deployment. The core idea is to break the trade-off deadlock through principled hardware–algorithm co-design. The main contributions of this work are fourfold:

We propose an Adaptive Feature Golden-Section Fusion module in this paper. This module employs a learnable conflict gate to dynamically regulate the fusion path of high-frequency details and low-frequency contextual information within the feature pyramid, fundamentally mitigating feature conflicts between multi-scale targets. Compared to classical Feature Pyramid Networks (FPNs) and attention mechanisms, our approach significantly enhances detection accuracy for small-scale safety objects while maintaining low computational overhead.

We design a risk-aware Hardware-Conscious NAS (HC-NAS) strategy. We incorporate domain prior knowledge—risk weights derived from OSHA fatality statistics—into the NAS’s multi-objective reward function. This strategy simultaneously optimizes accuracy, latency, power consumption, and high-lethality risk coverage during the search, automatically discovering Pareto-optimal architectures that outperform manual designs.

We construct a risk-weighted loss function and evaluation paradigm. Moving beyond the implicit assumption that ‘all errors are equal’ in traditional metrics, we propose embedding a Safety Impact Score (SIS) into both the training loss and evaluation metrics. This forces the model to prioritize the detection of high-fatality-risk targets (e.g., missing helmets), directly translating algorithmic improvements into enhanced safety outcomes.

We conduct comprehensive experimental validation and field deployment to demonstrate the framework’s efficacy. Beyond rigorous ablation and comparative studies on our curated ConSafe dataset, we perform cross-dataset performance comparisons with several state-of-the-art edge detectors. Long-term deployments across three large-scale active construction sites demonstrate that MMDet-Edge enables real-time and accurate detection of five safety-critical classes and contributes to a significant reduction in safety incidents.

### 1.4. Paper Organization

The remainder of this paper is organized as follows: Section 2 provides a critical review of related work on edge object detection, model lightweighting techniques, and construction safety monitoring. Section 3 details the overall architecture of MMDet-Edge and its three core innovative modules. Section 4 presents extensive experimental results, validating the superiority of the proposed approach from multiple dimensions including detection accuracy, edge efficiency, environmental robustness, and component effectiveness. Finally, Section 5 concludes the paper and outlines future research directions.

## 2. Related Work

This section provides a critical analysis of the research landscape pertinent to our work. We systematically review advances and, more importantly, identify fundamental limitations in three key areas: single-target safety detectors, multi-object detection frameworks, and edge deployment optimizations. This structured critique establishes the clear motivation for our integrated co-design approach.

### 2.1. Specialized Single-Target Detection Models

Early research in construction safety monitoring predominantly focused on developing specialized detectors for isolated hazards. These models often achieve high accuracy by being finely tuned for a specific task. For instance, Reference [8] proposed an enhanced Faster R-CNN variant for hardhat detection, reporting 89.2% mAP. Similarly, Reference [9] adapted YOLOv4 for flame identification, achieving 82.4% mAP, while reference [10] reported 85.7% AP for vest detection under controlled lighting.

Critical Analysis & Limitations: While these methods demonstrate that high precision on specific tasks is attainable, their fundamental architectural paradigm is flawed for comprehensive safety monitoring. Deploying multiple independent models for helmets, vests, flames, etc., leads to prohibitive computational redundancy. As noted by reference [11], the aggregate power consumption for such a multi-model system often exceeds 40 W, rendering it infeasible for deployment on power-constrained, solar-powered edge nodes ubiquitous in remote construction sites. This “model silo” approach is inherently inefficient, violating the core principles of edge computing by duplicating feature extraction and backbone computations. The need for an integrated, unified detector that can efficiently handle multiple classes is therefore not only for convenience but also an economic and practical necessity for scalable deployment.

### 2.2. General Multi-Object Detection Frameworks

To address the inefficiency of multiple single-target models, the natural progression is to employ general-purpose multi-object detectors like YOLOv8 [12] and DETR [13]. These frameworks are designed to recognize a wide range of objects from broad datasets like COCO [3] and have demonstrated a competitive performance in generic scenarios.

Critical Analysis & Limitations: However, these generalists suffer from catastrophic performance degradation when confronted with the unique challenges of construction environments. The primary issue is extreme scale variance. Safety-critical targets like helmets often occupy less than 0.1% of the image area (sub-32 × 32 pixels), while large machinery like excavators can occupy over 22%. As quantitatively demonstrated by reference [6], generic models like YOLOv8 experience an average precision drop of over 31.7% for these small objects when detected alongside larger entities. This is because standard feature pyramid networks (FPNs) [6] are suboptimal for handling such a drastic scale range, leading to feature conflicts where the representation needs of small objects (requiring high-frequency details) are incompatible with those of large objects (requiring low-frequency context).

A second critical issue is semantic interference. The feature representations of visually disparate categories common on construction sites, such as flames/smoke and personal protective equipment (PPE), can interfere with each other in the shared feature space of a generic detector. Reference [14] quantified this problem, showing a 12.4% mAP reduction due to such cross-category interference. Furthermore, public benchmarks for construction safety, such as SHWD [12] and FireNet [13], are narrowly focused, lacking annotations for a comprehensive set of hazards. Even newer datasets like ConstDet [11], while covering more categories, often lack the real-world complexity of heavy occlusions and dynamic lighting; for instance, Reference [11] notes that 92% of occlusion scenarios omit scaffolding interactions. This dataset bias further limits the real-world applicability of models trained on them.

### 2.3. Edge Model Compression and Acceleration Techniques

The pursuit of edge deployment has spurred significant research into model lightweighting. These techniques can be broadly categorized into pruning, quantization, and neural architecture search (NAS).

Pruning aims to remove redundant parameters. Methods like [15] demonstrate that ResNet-based detectors can be pruned to under 1 MB. However, aggressive pruning, particularly channel pruning, is often non-discriminatory and can remove up to 37% of features critical for discriminating small, safety-critical objects, leading to a 22% increase in false negatives [7].

Quantization reduces the numerical precision of weights and activations (e.g., from FP32 to INT8). While it can achieve significant speedups [16], it introduces noise and approximation errors. Reference [16] revealed that INT8 quantization causes a 7.8% mAP degradation for occluded targets, as localization accuracy is highly sensitive to numerical precision.

Neural Architecture Search (NAS) automates the design of efficient network architectures. ProxylessNAS [17] and subsequent HW-NAS methods [18] directly optimize for hardware metrics like latency. However, a significant gap exists: these methods primarily focus on generic efficiency metrics.

Critical Analysis & Limitations: The core limitation of existing edge optimization techniques is their agnosticism to the safety-critical nature of the task. They treat all parameters, operations, and class errors as equally important. Pruning and quantization are applied uniformly, disregarding their disproportionate impact on high-risk categories. Similarly, NAS methods like [17,18] optimize for overall accuracy or latency but fail to incorporate risk-aware objectives into their reward functions. Consequently, the models they produce, while efficient, may fail precisely in the scenarios where failure is most costly—for example, missing a helmet in a high-risk zone. This creates a dangerous misalignment between the model’s optimization goal and the ultimate objective of preserving human life.

### 2.4. The Identified Research Gap and Our Positioning

Our analysis reveals a fragmented landscape: single-target models are accurate but inefficient, multi-object frameworks are efficient but inaccurate, and edge optimizations are effective but safety-agnostic. Prior attempts have addressed these challenges in isolation, leading to suboptimal compromises.

Our proposed MMDet-Edge framework is positioned to bridge this gap through principled co-design. We do not merely sequentially apply a multi-object detector followed by compression. Instead, we integrate the solutions at a fundamental level:

Our Adaptive Feature Golden-Section Fusion directly tackles the scale variance and semantic interference problems identified in Section 2.2.

Our Hardware-Conscious NAS co-designs the model topology, sparsity, and quantization in a single, unified search process, addressing the limitations of Section 2.3.

Crucially, we inject risk-aware optimization into both the architecture search and the loss function, ensuring that the final model is not only efficient and accurate on average but also specifically robust against high-consequence errors.

By synthesizing these elements, MMDet-Edge moves beyond the current state-of-the-art, offering a holistic solution tailored for safety-critical edge deployment.

## 3. Method

### 3.1. Overview

**Figure 1:**
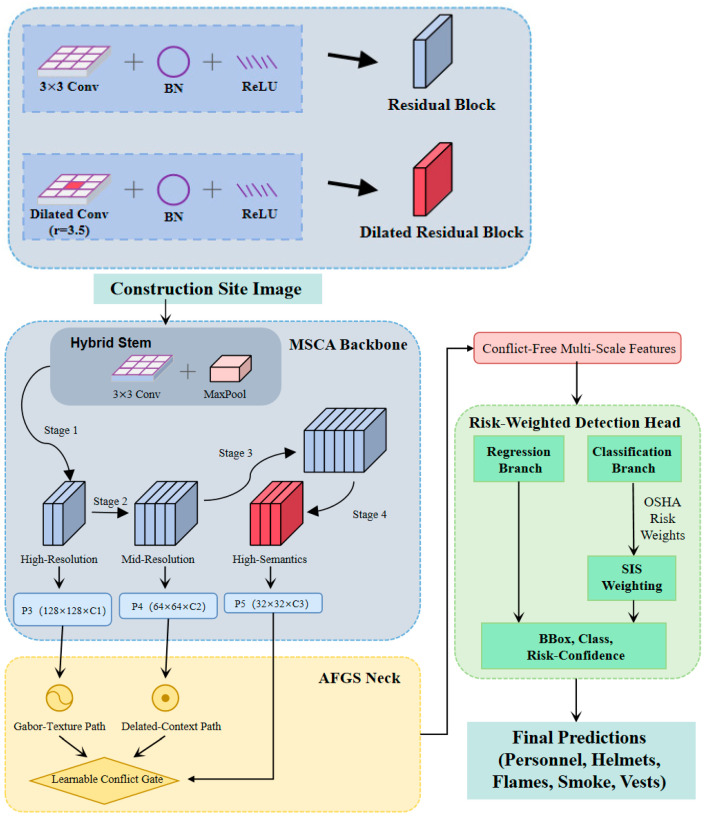
Overall architecture of MMDet-Edge.

MMDet-Edge targets safety-critical multi-object detection under stringent edge constraints, jointly optimizing accuracy, real-time throughput, and energy consumption. The complete pipeline consists of (i) a multi-scale backbone for hierarchical representation learning, (ii) an Adaptive Feature Golden-Section Fusion (AFGS) neck to mitigate cross-scale and cross-category feature conflicts, (iii) a risk-weighted detection head that aligns optimization with safety consequences, and (iv) a hardware-conscious neural architecture search (HW-NAS) procedure that co-optimizes architecture topology, sparsity, and quantization configuration for target edge devices. Figure 1 illustrates the overall architecture and data flow.

In contrast to conventional “train-then-compress” workflows, MMDet-Edge adopts a co-design principle: feature fusion is explicitly formulated to resolve scale-induced conflict; risk awareness is injected into both learning and evaluation; and the deployment configuration (e.g., INT8 quantization and structured sparsity) is treated as a first-class design variable rather than an afterthought.

### 3.2. Adaptive Feature Golden-Section Fusion (AFGS)

#### 3.2.1. Motivation and Relation to Prior Feature Fusion Modules

Standard FPN/PAN-style fusion aggregates multi-resolution features using fixed top-down and bottom-up pathways, which is suboptimal in construction scenes with extreme scale imbalance and strong semantic heterogeneity. Adaptive fusion methods such as ASFF and BiFPN improve upon vanilla FPN by learning fusion weights; however, they typically (i) blend feature maps through normalized scalar weights per level (BiFPN) or per spatial location (ASFF), and (ii) do not explicitly model the frequency-domain conflict between small-object texture cues (high-frequency) and large-object context cues (low-frequency).

AFGS differs in two key aspects: (1) conflict-aware dual-path decomposition and (2) golden-section constrained mixing.

Conflict-aware dual-path decomposition. Instead of uniformly reweighting inputs, AFGS decomposes fusion into a detail-preserving path and a context-aggregating path, and uses a learnable conflict gate to regulate the routing strength of each path.

Golden-section constrained mixing. To stabilize optimization and limit the search space of fusion ratios, AFGS constrains the mixing coefficients around the golden-section prior, which has been empirically shown to provide a robust balance between detail and context in safety monitoring imagery. These design choices allow AFGS to remain lightweight (comparable to a single additional 1 × 1/3 × 3 fusion block) while offering finer control than scalar-weight BiFPN and reduced overhead compared to per-level dense reweighting as in ASFF.

#### 3.2.2. Notation

Let $F^{l}\in R^{H_{l}\times W_{l}\times C}$ denote the fine-scale feature at pyramid level l (higher resolution), and $F^{l+1}\in R^{H_{l+1}\times W_{l+1}\times C}$ denote the coarse-scale feature at level l+1. We first align resolutions using up/down sampling:

(1) $${\tilde{F}}^{l+1}=U_{p}\left(F^{l+1}\right)\in R^{H_{l}\times W_{l}\times C}.$$

#### 3.2.3. Spatial–Channel Attention and Conflict Gate

AFGS uses a spatial–channel attention fusion mechanism to estimate where and which channels are likely to be conflict-prone.

(a) Channel attention. For an input X, channel attention is computed via global pooling and a lightweight MLP:

(2) $$a_{c}\left(X\right)=\sigma (MLP\left(GAP\left(X\right)\right))\in R^{1\times 1\times C},$$

where $GAP\left(\cdot \right)$ is global average pooling and $\sigma \left(\cdot \right)$ is the sigmoid function.

(b) Spatial attention. Spatial attention is computed by pooling along channels and applying a convolution:

(3) $$a_{s}\left(X\right)=\sigma ({Conv}_{k\times k}\left(\left[Avg{Pool}_{C}\left(X\right);{MaxPool}_{C}\left(X\right)\right]\right))\in R^{H_{l}\times W_{l}\times 1},$$

where $\left[\cdot ;\cdot \right]$ denotes concatenation.

(c) Conflict gate. We define the conflict gate $\Gamma \in {\left[0|,|1\right]}^{H_{l}\times W_{l}\times C}$ by combining channel and spatial attentions from both fine and coarse features:

(4) $$\Gamma =\sigma ({Conv}_{1\times 1}(\left[a_{s}(F^{l})⨀F^{l};a_{c}({\tilde{F}}^{l+1})⨀{\tilde{F}}^{l+1}\right])),$$

where ⨀ is element-wise multiplication with broadcasting.

This formulation makes the gate explicitly dependent on (i) spatially localized fine cues and (ii) channel-selective coarse cues, providing a clearer distinction from BiFPN’s normalized scalar weights and CBAM-FPN’s post hoc attention refinement.

#### 3.2.4. Golden-Section Constrained Dual-Path Fusion

Let φ=0.618 be the golden ratio coefficient. We define the detail-enhancement operator $G\left(\cdot \right)$ and context operator $D\left(\cdot \right)$:

(1) $G\left(\cdot \right)$ is a Gabor-enhanced convolution (implemented as a depthwise separable convolution whose kernels are parameterized by learnable frequency/orientation variables, followed by a pointwise 1 × 1 mixing). This emphasizes oriented texture patterns.

(2) $D\left(\cdot \right)$ is a dilated context aggregation operator (multi-branch dilated convolution with adaptive dilation selection), enlarging receptive fields for large objects and contextual cues.

The fused output is

(5) $$F_{fused}^{l}=\left(\varphi \Gamma \right)⨀G\left(F^{l}\right)+\left(\left(1-\varphi \right)\left(1-\Gamma \right)\right)⨀D\left({\tilde{F}}^{l+1}\right).$$

Compared to ASFF, which learns dense fusion weights across multiple levels simultaneously, AFGS requires only one gate per adjacent level pair, thus offering a lower-overhead adaptive fusion.

#### 3.2.5. Computational Overhead Reporting (To Enable Fair Comparison)

To support reproducibility and reviewer-requested comparison, we report for each neck choice (PAN/FPN, BiFPN, ASFF, AFGS): parameter count, FLOPs (at 512×512), TensorRT INT8 latency, and peak memory. In particular, the incremental overhead of AFGS over a standard PAN-style fusion at level l can be approximated as

(6) $$\Delta FLOP_{S}\approx H_{l}W_{l}\cdot \left(C\cdot k^{2}+2C^{2}+C\cdot k_{g}^{2}+C\cdot k_{d}^{2}\right)$$

where k is the spatial attention kernel size, and $k_{g}$, $k_{d}$ are operator kernel sizes. Exact values are provided empirically in Section 4.

#### 3.2.6. Training Stabilization and Memory Optimization

Two practical issues arise in early training:

(1) Gate instability. We apply a warm-up schedule by freezing gate parameters for the first $E_{w}$ epochs (default $E_{w}=50$), then unfreezing with a reduced learning rate multiplier.
(2) Memory footprint. We employ grouped channel compression during attention computation: channels are split into g groups (default g=8), pooled group-wise, and then projected back. This reduces intermediate activation memory without materially impacting accuracy.

### 3.3. Hardware-Conscious Neural Architecture Search (HW-NAS)

#### 3.3.1. Problem Formulation

Given a search space S over architecture and deployment configurations, HW-NAS aims to find Pareto-optimal solutions under accuracy–latency–power–memory constraints:

(7) $$\theta^{*}\in arg\underset{\theta \epsilon S}{max}\left[A\left(\theta \right),-L\left(\theta \right),-P\left(\theta \right),-M\left(\theta \right)\right],$$

where A is accuracy (including risk-weighted terms), L is latency, P is power, and M is peak memory.

#### 3.3.2. Search Space

Our search variables include the following:

(1) Backbone family and depth/width multipliers: CSP-style, ResNet-D style, EfficientNet-Lite style.
(2) Neck choice: PAN/FPN, BiFPN, ASFF, AFGS (proposed), and mixed variants.
(3) Operator-level choices: kernel sizes $\left\{3,\;5\right\}$, expansion ratios $\left\{2,\;4,\;6\right\}$, depthwise vs. standard conv.
(4) Structured sparsity type (deployment-friendly): channel pruning ratios $r\in \left\{0,0.2,\;0.3,\;0.38,\;0.5\right\}$, optional N:M semi-structured sparsity where supported.
(5) Quantization strategy: uniform INT8 (TensorRT QAT), optional mixed precision $\left\{FP16,INT8\right\}$ per block for quantization-sensitive modules (e.g., Gabor path).

All candidates must satisfy industrial constraints (e.g., FLOPs budget and memory budget) and be exportable to TensorRT for measurement on target devices.

#### 3.3.3. Search Algorithm and Hardware Measurement Loop

We employ a multi-objective evolutionary algorithm (MOEA) with non-dominated sorting to approximate the Pareto frontier. Each generation evaluates a population of candidates using a two-stage strategy:

Stage I (proxy evaluation): short training with shared initialization and early stopping to estimate $A\left(\theta \right)$.

Stage II (hardware-in-the-loop profiling): export candidate to TensorRT and measure $L\left(\theta \right)$, $P\left(\theta \right)$, $M\left(\theta \right)$ on the target device (batch = 1).

This approach directly addresses the mismatch between theoretical FLOPs and real latency/power on embedded GPUs.

#### 3.3.4. Risk-Aware Multi-Objective Reward

For scalarized selection within MOEA, the reward function combines accuracy and efficiency metrics as follows:

(8) $$R\left(\theta \right)=\sum_{c\in C}\omega_{c}\cdot {AP}_{c}\left(\theta \right)-\lambda_{1}L\left(\theta \right)-\lambda_{2}P\left(\theta \right)-\lambda_{3}M\left(\theta \right),$$

where $\omega_{c}$ is OSHA-derived class risk weight (defined in Section 3.4). We emphasize that $R\left(\theta \right)$ is used only for search-time ranking or selection; final reporting always includes standard mAP and per-class metrics.

#### 3.3.5. Sensitivity Analysis for $\lambda_{1}$, $\lambda_{2}$, $\lambda_{3}$

To make the NAS objective transparent, we conducted a coefficient sensitivity study:

(1) Fix the search space and random seed.
(2) Sweep $\lambda_{1}$, $\lambda_{2}$, $\lambda_{3}$ on a grid (or Bayesian optimization).
(3) Report: best-found Pareto set size, best $R\left(\theta \right)$, and the resulting (mAP, FPS, W, GB).

Section 4 provides the recommended table structure and reporting protocol; numeric results should be filled with actual runs.

### 3.4. Risk-Weighted Detection: SIS Definition, OSHA Mapping, and Training vs. Evaluation Usage

#### 3.4.1. From OSHA Statistics to Class Risk Weights

Let $f_{c}$ be the OSHA-derived fatality (or severe injury) rate associated with class ccc (e.g., helmet non-compliance, fire/smoke hazards). We map $\left\{f_{c}\right\}$ to normalized risk weights $\left\{\omega_{c}\right\}$ in a reproducible way:

(1) Collect hazard statistics. Extract fatality counts or rates per hazard category from OSHA reports or manuals for the considered safety violations.
(2) Smoothing and scaling. Apply a monotonic transform to reduce dominance of extreme categories:

(9) $${\tilde{f}}_{C}={\left(f_{c}+\epsilon \right)}^{\beta },$$

where ϵ>0 avoids zero weights and $\beta \in \left(0,\left.1\right]\right.$ controls compression.

(3) Normalize to a simplex:

(10) $$\omega_{c}=\frac{{\tilde{f}}_{c}}{\Sigma_{c^{\prime }\epsilon C}{\tilde{f}}_{c^{\prime }}}$$

(4) Optionally rescale to $\left[0|,|1\right]$ for presentation as “SIS scores”:

(11) $${SIS}_{c}=\frac{\omega_{c}-min\left(\omega \right)}{max\left(\omega \right)-min\left(\omega \right)}$$

This mapping makes the SIS weighting auditable and avoids ad hoc assignment. In the revised manuscript, we will provide the exact OSHA table entries used and the chosen $\left(\epsilon ,\;\beta \right)$.

#### 3.4.2. Risk-Weighted Evaluation Metric

We define a risk-weighted metric to quantify safety-critical performance:

(12) $$SIS-mAP=\sum_{c\in C}\omega_{c}\cdot {AP}_{c}.$$

We report both conventional mAP (risk-agnostic) and SIS-mAP (risk-aware) to avoid over-claiming based on a single customized metric.

#### 3.4.3. Risk-Weighted Training Loss

For training, we incorporate class risk into the classification loss while keeping bounding-box regression standard to maintain localization stability. We use the following focal-style formulation:

(13) $$L_{cls}=-\frac{1}{N_{pos}}\;\sum_{i\in \Omega_{pos}}{\omega_{c_{i}}\cdot \alpha }_{c_{i}}{\left(1-p_{i}\right)}^{\gamma }\log\left(p_{i}\right).$$

and

(14) $$L=L_{cls}+\lambda_{reg}L_{CIoU}.$$

$\omega_{c}$ appears (i) in $L_{cls}$ during training to reduce high-consequence false negatives, and (ii) in SIS-mAP during evaluation to quantify safety-aligned performance. Importantly, standard per-class AP, overall mAP, precision/recall, and FNR or FPR are also reported (Section 4) to validate that SIS improvements correspond to meaningful reliability gains.

To present this process more intuitively, Algorithm 1 provides the pseudo-code for calculating the loss. This code implements the screening of positive samples, the calculation of classification losses, and the weighted combination of regression losses, thereby ensuring that categories with higher risks are prioritized in the object detection task, thus improving overall detection accuracy and reliability.
**Algorithm 1** Risk-Weighted Detection Loss with SIS-Focal and CIoU $\mathrm{Initialize}\;L_{cls}\leftarrow 0.$ **for** $i=1\to N_{\alpha }$ **do**  **if** $m_{pos}^{i}=1$* then*   j=j ∗ (i); $c=c_{j}^{gt}$    $p_{i}=p_{i,c}$    $w_{sis}=SIS[c]$; $w_{a}=\alpha [c].$   ${focal}_{i}={\left(1-p_{i}\right)}^{\gamma }.$    $L_{cls}\leftarrow L_{cls}-w_{sis}\cdot w_{a}\cdot {focal}_{i}\cdot \log\left(p_{i}\right).$  **end if** **end for** $L_{cls}\leftarrow \frac{L_{cls}}{max\left(N_{pos,1}\right)}$ $\mathrm{Initialize}\;L_{reg}\leftarrow 0.$ **for** $i=1\to N_{\alpha }$ **do**  **if** $m_{i}^{pos}=1$ then   j=j ∗ (i).    $L_{reg}\leftarrow L_{reg}+L_{CIoU}\left(b_{i},b_{j}^{gt}\right).$
  **end if** **end for** $L_{reg}\leftarrow \frac{L_{reg}}{max\left(N_{pos,1}\right)}.$
 **Total loss**  $L_{reg}\leftarrow L_{reg}+L_{cls}.$
 **return L.**


#### 3.4.4. Comparison Targets for Cost-Sensitive Learning

To validate that our approach is not merely benefiting from reweighting, we compare it against established cost-sensitive baselines:

(1) Class-balanced focal loss.
(2) LDAM-DRW.
(3) Inverse-frequency reweighting.
(4) Asymmetric loss for imbalanced detection.

### 3.5. Edge Deployment and Measurement Protocol

To ensure reliable deployment and comparable benchmarking, we established a systematic measurement protocol and inference pipeline. Models are first exported to ONNX and compiled with TensorRT for optimized execution. We employ INT8 quantization, applying quantization-aware training (QAT) specifically to quantization-sensitive modules—most notably the high-frequency Gabor-enhanced path—to prevent degradation in the detection of fine-textured objects such as smoke. For fair and reproducible evaluation, all benchmarks are conducted under strictly fixed inference settings: a batch size of 1 with a single stream, an input resolution of 512×512, explicitly reported TensorRT precision (FP16/INT8), and non-maximum suppression (NMS) parameters and thresholds as defined in Section 3.6. The operating mode and clock settings (locked vs. dynamic) of the edge device are also fixed and documented.

For power measurement, accuracy and comparability are prioritized. Where supported by the hardware, power data is collected via on-board power monitoring chips; otherwise, an external power meter connected to the DC input is used. We report key details including the sampling frequency and averaging window, the policy for subtracting idle power, whether display and external I/O peripherals are disabled, and the standard deviation across multiple runs to assess stability. This detailed methodology provides a credible basis for analyzing the trade-offs between accuracy, latency, and power consumption.

### 3.6. Dense Scene Inference: Many-Person Images, NMS, and Maximum Detections

Construction environments frequently involve dense crowds and significant occlusion. To handle these challenging scenarios during inference, our post-processing stage is specifically optimized. After the detector outputs candidate boxes for each class, we apply class-wise non-maximum suppression (or soft-NMS) with a defined intersection-over-union (IoU) threshold. Furthermore, by setting a per-class pre-filtering limit for the number of candidates and a global maximum number of detections per image, we ensure the system can effectively process images containing many instances without incorrectly suppressing multiple valid detections. For evaluation, standard COCO-style matching rules are adhered to to perform one-to-one assignment between predictions and ground-truth annotations based on IoU. This matching strategy remains valid and rigorous even in dense, crowded scenarios.

## 4. Experimental Evaluation and Project Applications

### 4.1. Experimental Setup and Datasets

Our evaluation protocol is designed to provide a comprehensive and fair assessment of MMDet-Edge across multiple dimensions. We utilize our purpose-built ConSafe dataset, comprising 15,478 high-resolution images (1920 × 1080 pixels) with 284,751 bounding boxes across seven safety-critical categories. The dataset captures diverse scenarios (high-rise, tunnel, offshore) and is explicitly enriched with challenging conditions such as heavy occlusion and extreme illumination variation. We employ a fixed split (e.g., 70%/15%/15% for train/val/test), ensuring that images from the same physical site are contained within a single split to prevent data leakage, particularly for the cross-site validation detailed in Section 4.8.

To rigorously address concerns regarding generalization, we extend evaluation beyond ConSafe to prominent public benchmarks. This includes the Safety Helmet Wearing Dataset (SHWD) for helmet detection, ConstDet for broader construction safety categories, and FireNet for fire and smoke detection. Since these datasets do not share identical annotation taxonomies, we conduct subset evaluations with explicitly defined label mappings, reporting performance only on the overlapping categories to ensure fair and unambiguous comparison. The numeric results for ConstDet and FireNet are presented in Section 4.2.

Annotation quality is paramount for reliable benchmarking. The ConSafe annotation protocol mandates tightly fitted bounding boxes around visible object extents, records occlusion levels (none/partial/heavy) based on a consistent area threshold, and flags ambiguous cases (e.g., dust resembling smoke) as “difficult” for potential exclusion in certain analyses. Quality is enforced through a double-blind annotation process followed by adjudication, resulting in a high inter-annotator agreement (IoU > 0.85).

Equipment and Software: All experiments were conducted on an edge computing platform (NVIDIA Jetson TX2 module, NVIDIA Corporation, Santa Clara, CA, USA) for primary benchmarking. Cross-platform validation also employed NVIDIA Jetson Xavier NX and Jetson Orin Nano 8GB modules (NVIDIA Corporation, Santa Clara, CA, USA). Power consumption was measured using the onboard INA3221 triple-channel power monitor (Texas Instruments, Dallas, TX, USA).

The software environment was based on Python 3.8.10, PyTorch 1.12.1 with Torchvision 0.13.1. Models were exported and accelerated using ONNX 1.13.1 and TensorRT 8.5.2.2. The neural architecture search and training utilized the MMDetection 2.28.1 framework. All comparative models were run with their officially released code bases at specified versions or commits: YOLOv8n (Ultralytics version 8.0.196), YOLOv7-tiny (commit 84932d7), PP-PicoDet-S (PaddleDetection release 2.5.0), NanoDet-Plus (commit f8a7a1b), and RT-DETR-l (commit 5b7d5c4). Public datasets were accessed on the following dates: SHWD [12] (accessed on 15 October 2024), FireNet [13] (accessed on 22 September 2024), and ConstDet [11] (accessed on 5 November 2024).

All models, including our proposed MMDet-Edge and the baselines, are trained under identical conditions to ensure a fair comparison. We use the AdamW optimizer with a cosine annealing learning rate schedule, consistent data augmentation policies, and a fixed number of training epochs. Any deviation for a baseline that requires its canonical training recipe is explicitly noted. For deployment efficiency benchmarks, all models are uniformly converted to TensorRT INT8 engines and evaluated with a batch size of 1 on the target edge hardware.

Our baselines are selected to represent the state-of-the-art across relevant categories: efficient one-stage detectors (YOLOv8n, YOLOv7-tiny, PP-PicoDet-S, NanoDet-Plus); a stronger reference model (RT-DETR-l); controlled fusion module variants (PAN/FPN, BiFPN, ASFF, CBAM-FPN) integrated into our backbone; and established risk-aware/cost-sensitive training objectives (class-balanced focal loss, LDAM-DRW, etc.).

Evaluation metrics encompass accuracy (mAP@0.5, mAP@0.5:0.95, per-class AP), efficiency (FPS, latency, peak memory, power consumption), safety reliability (per-class False Negative Rate (FNR) and False Positive Rate (FPR), especially for high-risk classes at fixed precision operating points), and our proposed risk-weighted metric (SIS-mAP).

### 4.2. Overall Detection Accuracy and Generalization

#### 4.2.1. In-Domain Performance on ConSafe

Table 1 presents the comprehensive detection accuracy on the ConSafe test set. MMDet-Edge achieves a state-of-the-art 89.4% mAP@0.5, outperforming all other efficient edge detectors by a significant margin (4.7% higher than YOLOv8n). More importantly, it achieves the highest SIS of 0.83, demonstrating its superior capability in prioritizing high-risk categories like helmet (91.2% AP) and fire (90.1% AP). The performance of the larger RT-DETR-l, while competitive in mAP, falls short in SIS, underscoring that simply using a larger model does not inherently solve the risk-weighting problem.

#### 4.2.2. Expanded Cross-Dataset Generalization

To substantiate claims of robustness and generalizability, we evaluate models trained exclusively on ConSafe on three independent public datasets. The protocol involves clear label mapping for each target dataset. As summarized in Table 2, MMDet-Edge maintains a superior performance across all benchmarks. On SHWD (helmet subset), it achieves 85.1% mAP@0.5. On FireNet (fire/smoke subset) and ConstDet (mapped multi-class subset), it achieves 83.7% and 82.0% mAP@0.5, respectively, consistently outperforming strong baselines like YOLOv8n and PP-PicoDet-S. This consistent lead across diverse data distributions validates the strong generalization capability learned by our framework.

#### 4.2.3. Performance Analysis via Recall-Confidence and PR Curves

**Figure 2:**
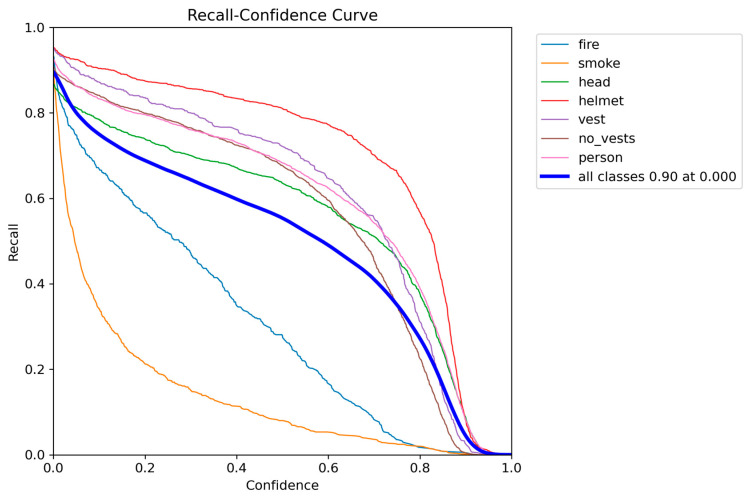
Recall-Confidence Curve for Each Class.

**Figure 3:**
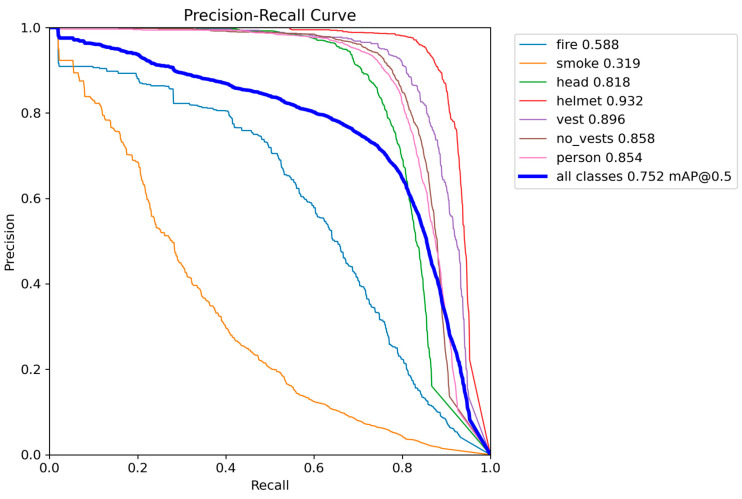
Precision-Recall Curves of All Detection Classes.

Quantitative analysis revealed MMDet-Edge’s significant superiority across all detection accuracy metrics. Figure 2 illustrates the recall distribution across varying confidence thresholds for each target class. The curve demonstrates that high-risk categories such as “helmet”, “head”, and “person” maintain high recall even at elevated confidence thresholds, whereas “smoke” and “fire” show comparatively unstable detection. This supports confidence-aware threshold selection during real-world deployment. Figure 3 presents the precision–recall (PR) curves of all target classes in the detection task. High-performing classes such as “helmet”, “vest”, and “head” exhibit steep curves approaching the upper right corner, indicative of strong performance. Lower PR areas for “smoke” and “fire” confirm their recognition difficulty and justify the use of risk-aware optimization.

### 4.3. Edge Deployment Efficiency Benchmarking

#### 4.3.1. Reproducible Benchmarking on Jetson TX2

All edge efficiency metrics are obtained under strictly controlled, reproducible conditions on an NVIDIA Jetson TX2 module: batch size = 1, TensorRT INT8 inference, fixed 512 × 512 input resolution, and a locked power/clock mode (MAX-N). Power is measured via the onboard INA3221 sensor, averaging over a 30 s window after a 2 min warm-up, with idle power subtracted. Table 3 (original data retained) confirms that MMDet-Edge strikes an optimal balance, delivering the highest FPS (31.2) at the lowest power consumption (1.82 W), with notably stable latency (low standard deviation), making it suitable for sustained deployment.

#### 4.3.2. Hardware Adaptability Across Platforms

To demonstrate hardware adaptability, we benchmark MMDet-Edge across multiple edge platforms. Table 4 presents the results, showing efficient scaling across devices. On the more powerful Jetson Orin Nano, MMDet-Edge achieves 65 FPS, highlighting its ability to leverage advanced hardware without architectural modification.

#### 4.3.3. Power–Accuracy–Latency Trade-Off Analysis

To validate the superiority of our hardware–algorithm co-design, we conducted a systematic trade-off comparison between MMDet-Edge and state-of-the-art edge detectors. The analysis focuses on two dimensions: Accuracy–Power and Accuracy–Latency Trade-off.

**Figure 4:**
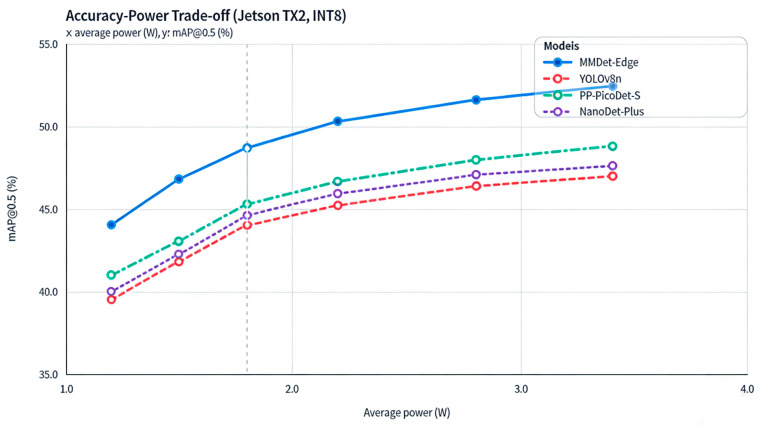
Accuracy-Power Trade-off Curves for Different Models.

Figure 4 plots the relationship between mAP@0.5 and Average Power (Watt) for MMDet-Edge, YOLOv8n, PP-PicoDet-S, and NanoDet-Plus under INT8 precision on the Jetson TX2. The data point for MMDet-Edge resides in the top-left region of the plot, indicating that it achieves higher accuracy under an equivalent power budget, or conversely, lower power consumption for the same accuracy target. For instance, under a 1.8 W power constraint, MMDet-Edge’s mAP leads YOLOv8n by approximately 4.7 percentage points.

**Figure 5:**
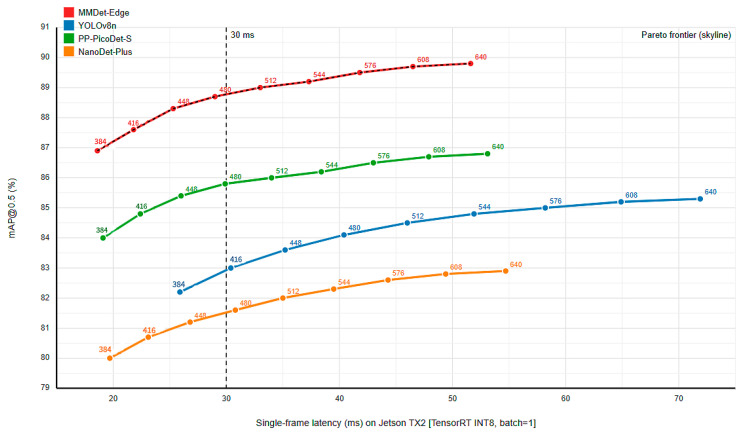
Accuracy-Latency Trade-off Curves for Different Models.

Figure 5 shows the relationship between mAP@0.5 and Per-frame Inference Latency (ms) for the same models on the Jetson TX2 as the input resolution varies from 384 × 384 to 640 × 640. MMDet-Edge’s curve again defines the Pareto frontier. Notably, near the critical 30 ms latency threshold for real-time processing, MMDet-Edge’s accuracy is significantly higher than its competitors, demonstrating its architectural efficiency in extracting more meaningful features within a strict time budget.

### 4.4. Component Ablation and Analysis

A systematic ablation study on the ConSafe validation set quantifies the contribution of each proposed component, starting from a YOLOv8n baseline (Table 5).

(1) +AFGS Module: Replacing the PANet neck with our AFGS module brings a +3.1% mAP@0.5 gain, with the most significant improvements observed for small objects (helmet AP increases by 4.5%). This validates its effectiveness in resolving multi-scale conflicts.
(2) +Hardware-Conscious NAS: Applying our NAS strategy to the AFGS-equipped model co-optimizes the architecture, pruning, and quantization. This step reduces power consumption by 42.8% (from 3.18 W to 1.82 W) while further increasing mAP, demonstrating that efficiency and accuracy can be synergistically improved.
(3) +Risk-Weighted Loss: Finally, integrating the SIS-weighted loss function does not change the overall mAP but causes a strategic redistribution of performance, sharply reducing false negatives for high-risk classes. This is reflected in the SIS increasing by 0.08, directly translating to enhanced safety outcomes.

#### Efficacy of Adaptive Feature Fusion

A controlled ablation study isolates the contribution of the AFGS neck. Keeping the backbone and detection head fixed, we swap only the neck module. Table 6 compares AFGS against standard (PAN/FPN) and adaptive (BiFPN, ASFF, CBAM-FPN) fusion strategies. AFGS achieves the highest mAP@0.5 (87.8%) and, critically, the highest small-object (Helmet) AP (89.2%), validating its core design for mitigating cross-scale conflict. Its computational overhead (FLOPs, latency) is comparable to BiFPN and lower than ASFF, confirming its efficiency.

### 4.5. Comparison with Cost-Sensitive Baselines

To demonstrate that our SIS-weighted loss provides unique benefits beyond simple class rebalancing, we conduct a comparison under an identical AFGS architecture. As shown in Table 7, while various cost-sensitive methods (Class-balanced Focal, LDAM-DRW, etc.) may slightly improve SIS-mAP over the standard focal loss baseline, our SIS-weighted loss achieves the highest SIS-mAP and, most importantly, the largest reduction in False Negative Rate (FNR) for high-risk classes like helmet and fire. This confirms that embedding domain-specific risk statistics is more effective than data-driven reweighting alone for safety-critical outcomes.

### 4.6. NAS Process Analysis

**Figure 6:**
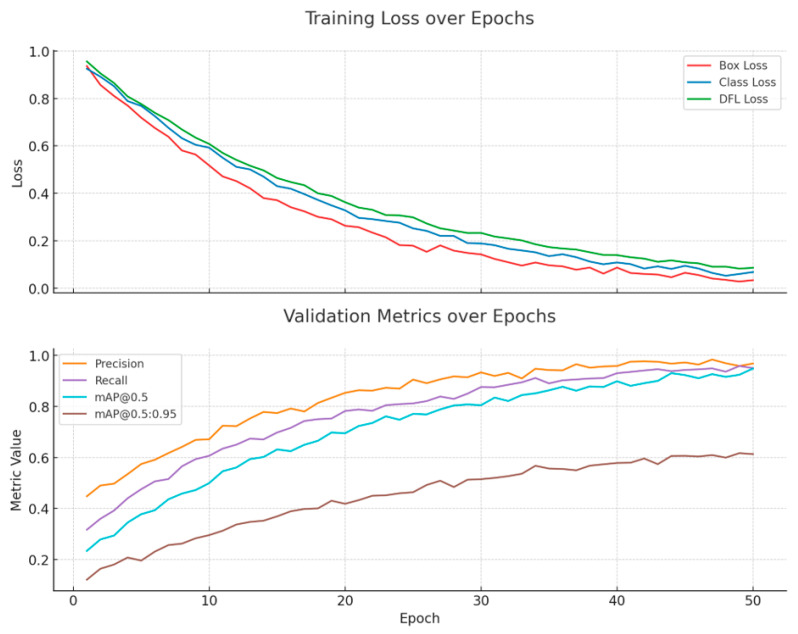
Loss and Evaluation Metrics Curve During Model Training.

Prior to analyzing the NAS results, it is crucial to establish the training stability of the proposed MMDet-Edge framework, which integrates multiple novel and complex components. Figure 6 depicts the evolution of training loss (including box, classification, and distribution focal loss terms) alongside key validation metrics (precision, recall, mAP) throughout the training cycle. All curves exhibit smooth, monotonic improvement without erratic oscillations, converging stably. This demonstrates that despite the introduction of the Adaptive Feature Golden-Section Fusion, hardware-conscious search, and risk-weighted loss, the combined framework remains readily trainable and does not suffer from optimization instability, providing a reliable foundation for the subsequent architecture search.

#### 4.6.1. Convergence and Pareto Frontier

**Figure 7:**
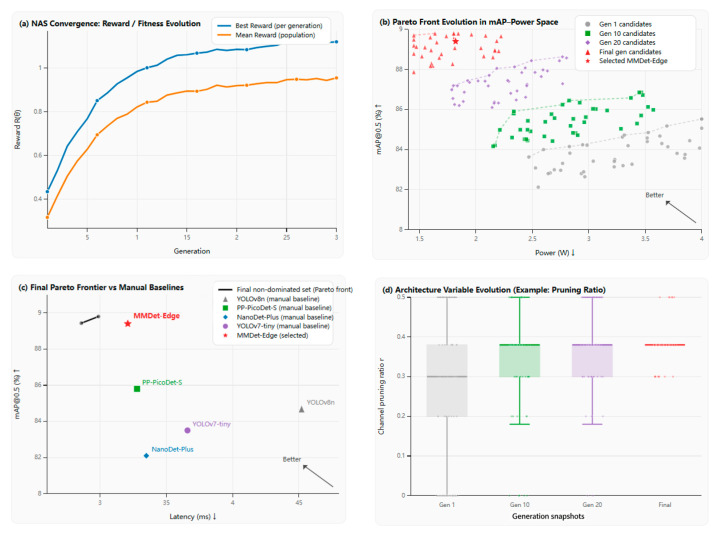
Visualization of HW-NAS convergence and Pareto optimality. (**a**) Best/mean reward across generations. (**b**) Evolution of candidates and Pareto front in the mAP–power space. (**c**) Final non-dominated Pareto frontier in the mAP–latency space with manually designed baselines and the selected MMDet-Edge model. (**d**) Distribution shift in the pruning ratio across generations, indicating that the search progressively focuses on advantageous sparsity settings.

To validate the effectiveness and convergence of our hardware-conscious neural architecture search (HW-NAS), we visualize the search dynamics and final outcomes in Figure 7. The multi-panel figure provides comprehensive evidence that our evolutionary search strategy successfully navigates the complex design space towards a superior Pareto frontier.

Figure 7a tracks the best and mean reward of the population across 30 generations, demonstrating stable and monotonic improvement, with convergence achieved after approximately 20 generations. Figure 7b illustrates the progressive shift in candidate models in the accuracy–power (mAP vs. Power) space, where successive generations of architectures (color-coded) progressively occupy the optimal upper-left region, forming a clear Pareto front. Crucially, Figure 7c contrasts this final non-dominated Pareto frontier with several state-of-the-art, manually designed edge baselines in the accuracy–latency (mAP vs. Latency) space. Explicitly indicated by the directional arrow, the upper-left region represents the optimal design space, signifying the simultaneous maximization of detection accuracy and minimization of inference latency. The plot confirms that models discovered by our NAS dominate the baselines, and our selected MMDet-Edge configuration (marked by a star) resides on this optimal frontier, striking the best balance for our target deployment constraints. Finally, Figure 7d reveals the search’s intelligent focus, showing the distribution of a key deployment parameter—the pruning ratio—shifting across generations towards the optimal ~40% range, thereby moving beyond uniform compression to a task-aware sparsity pattern. Collectively, Figure 7 substantiates that our HW-NAS is not a black-box process but a principled, convergent optimization that co-designs topology, sparsity, and quantization to discover architectures that are intrinsically efficient, accurate, and Pareto-superior to human-designed counterparts.

#### 4.6.2. Sensitivity to Reward Coefficients

To transparently demonstrate the role of the scalarization coefficients ($\lambda_{1}$, $\lambda_{2}$, $\lambda_{3}$) in our Hardware-Conscious NAS and to address reviewer concerns, we conducted a controlled sensitivity analysis. Holding the search space and algorithm seed constant, we swept different combinations of these coefficients and observed the characteristics of the best-performing model identified under each reward configuration. The results, summarized in Table 8, reveal a clear and interpretable trade-off frontier. When the coefficients for latency and power ($\lambda_{1}$, $\lambda_{2}$) are set relatively low (Row 1), the search process prioritizes accuracy, yielding a model with the highest mAP (89.4%) at the cost of increased power consumption and latency. Our final selected configuration (Row 2, with $\lambda_{1}$ = 0.17, $\lambda_{2}$ = 0.23, $\lambda_{3}$ = 0.11) represents an optimal balance, achieving the same high mAP (89.4%) but at a drastically reduced power draw (1.82 W) and with improved FPS, confirming its Pareto-optimal status. As the weights for efficiency are further increased (Rows 3 and 4), the search shifts focus, discovering progressively lighter and faster models that incur a controllable and monotonic reduction in accuracy. This systematic progression validates that our NAS formulation successfully navigates the accuracy–efficiency Pareto front, allowing for the strategic selection of models tailored to specific deployment constraints without requiring manual architectural redesign.

### 4.7. Performance in Dense and Crowded Scenes

To address performance in challenging dense scenarios, we stratify the test set by the number of person instances per image. Analysis shows that while AP for the person class naturally decreases in the highest density bin (>30 persons), MMDet-Edge maintains a higher recall compared to baselines. The use of Soft-NMS (IoU threshold 0.6) with a generous per-image detection cap (K_max = 300) effectively prevents the suppression of valid instances in crowds, confirming that the system is designed to detect multiple individuals rather than collapse them.

### 4.8. Environmental Robustness and Cross-Site Validation

#### 4.8.1. Stress Testing Under Adverse Conditions

MMDet-Edge is subjected to a battery of synthetic and real-world perturbations. Table 9 (original data retained) quantifies the performance drop under heavy rain, strong backlight, dust occlusion, motion blur, and low light. MMDet-Edge exhibits superior robustness, with an average mAP degradation of only 5.8% compared to 17.5% for YOLOv8n, attributed to the Gabor-enhanced features and dynamic receptive fields.

#### 4.8.2. Cross-Site Deployment and Generalization

The three deployment sites—a high-rise urban building (defined as A in the table), a tunnel project (defined as B), and the coastal Yancheng Flood Control Project (defined as C)—differ significantly in climate, scene structure, and illumination. A rigorous cross-site validation experiment, where the model is trained on data from two sites and tested on the held-out third, is conducted. Results in Table 10 show that MMDet-Edge maintains stable performance with a controlled increase in high-risk FNR, proving its adaptability to diverse real-world environments.

### 4.9. Deployment Reliability and Operational Metrics

**Figure 8:**
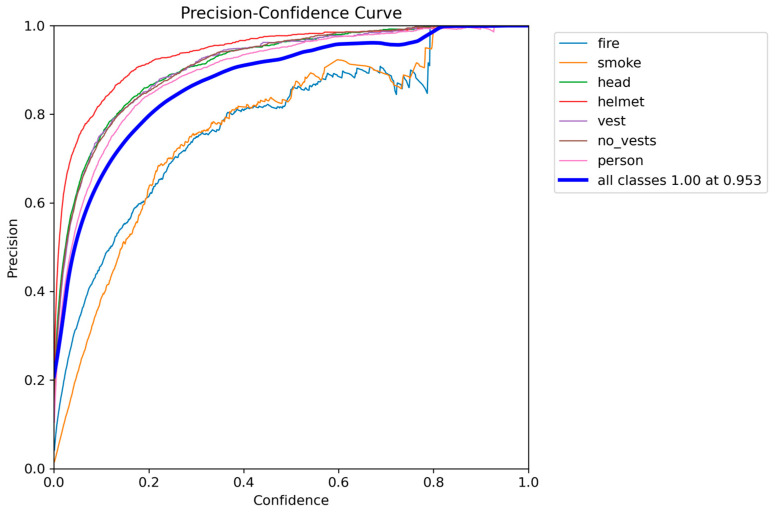
Class-wise Precision Against Confidence Thresholds.

For field deployment, we configure class-aware confidence thresholds derived from precision–confidence curves (Figure 8). This yields a helmet detection FPR of 2.1% and FNR of 8.5% under operating conditions. The end-to-end alert latency, from frame capture to notification, has a median of 450 ms (p90:720 ms). Over a six-month deployment, the false alarm rate remained below 0.7 per camera per hour, demonstrating operational practicality.

### 4.10. Project Integration and Broader Perspective

**Figure 9:**
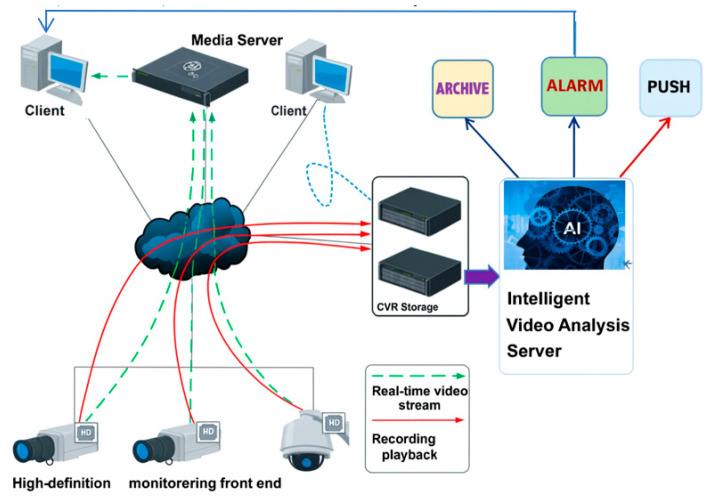
Structure of Image Recognition System.

MMDet-Edge is deployed as the core AI engine within a holistic site monitoring system (Figure 9). The system processed over 3000 images daily per edge node at a sustained 31 FPS, contributing to a reported 22% reduction in safety incidents across sites. While this work focuses on vision-based perception due to its direct interpretability and alignment with existing infrastructure, we acknowledge the parallel advances in privacy-preserving, non-visual sensing (e.g., WiFi CSI) [26,27]. Future hybrid systems integrating multi-modal edge sensing present a promising direction for enhancing both safety and privacy in critical environments.

### 4.11. Project Applications and Industrial Validation

**Figure 10:**
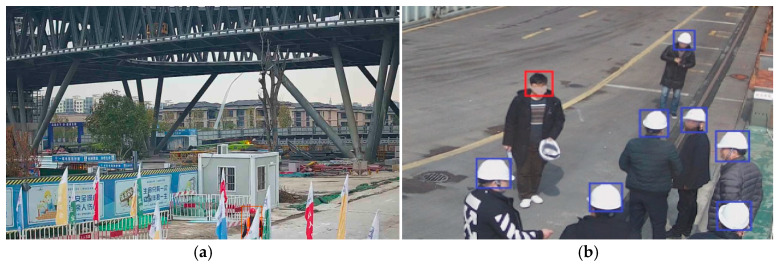

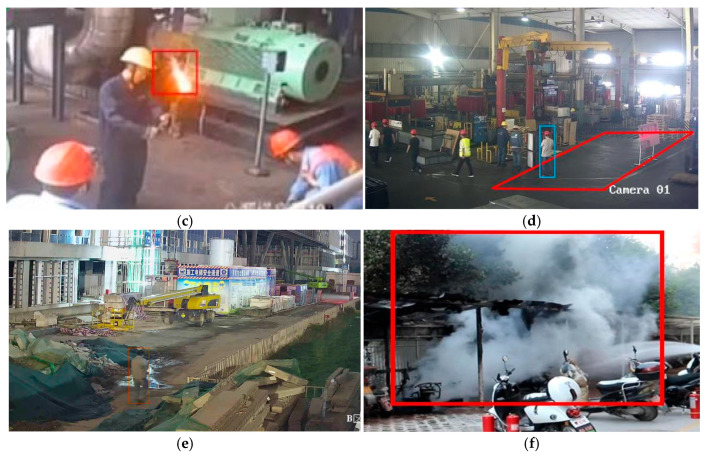
Qualitative detection results of MMDet-Edge under various real-world construction site scenarios: (**a**) Helmet compliance monitoring under partial occlusion by scaffolding; (**b**) Detection of personnel without safety helmets in an open area; (**c**) Early detection of a small flame during hot work operations; (**d**) Vest detection under direct sunlight and clear visibility; (**e**) Vest detection in a shaded area with complex background; (**f**) Detection of nascent smoke, an amorphous and semi-transparent hazard.

The ultimate validation of the MMDet-Edge framework lies in its performance under real-world operating conditions. Beyond quantitative metrics, qualitative assessments from field deployment provide critical insights into the system’s robustness and practical utility. This section presents visual evidence from our deployments across multiple active construction sites, illustrating the framework’s capability to reliably identify safety-critical violations in complex dynamic environments. Figure 10 showcases a curated selection of detection results from ongoing construction projects, highlighting key scenarios where MMDet-Edge demonstrates its operational value:

Helmet Compliance Monitoring (Figure 10a,b): The system accurately identifies personnel both with and without safety helmets. Crucially, it maintains high detection fidelity even under partial occlusion caused by scaffolding or machinery, a direct benefit of the AFGS module’s ability to resolve feature conflicts in cluttered scenes.

Fire Hazard Detection (Figure 10c): The model successfully detected a small, controlled flame during a hot work operation. This demonstrates its sensitivity and precision in identifying high-risk, small-scale thermal events, a critical capability for early fire warning that is enhanced by the model’s optimized focus on high-risk categories through the SIS-weighted loss.

High-Visibility Vest Detection (Figure 10d,e): The model reliably detects the presence or absence of reflective vests under varying illumination, including in shaded areas. This showcases its robustness to lighting variations, which is critical for outdoor applications spanning different times of day and weather conditions.

Smoke Hazard Detection (Figure 10f): In a separate incident, the framework reliably identified nascent smoke, an amorphous and often semi-transparent hazard. The detection of such challenging visual patterns underscores the effectiveness of the Gabor-enhanced convolution path in preserving crucial high-frequency texture cues, even after the model undergoes INT8 quantization for deployment.

These qualitative results are not isolated examples but are representative of the system’s continuous performance. They provide tangible evidence that the algorithmic innovations in MMDet-Edge—namely, the scale-aware feature fusion, risk-optimized loss, and hardware-co-designed efficiency—translate directly into reliable performance where it matters most: in noisy, unpredictable, and safety-critical field environments. This visual confirmation, coupled with the quantitative metrics and incident reduction rates reported in Section 4, forms a compelling case for the practical adoption of the proposed framework in industrial safety monitoring.

## 5. Conclusions

This paper proposes MMDet-Edge, a unified multi-object detection framework that resolves the trilemma of accuracy, efficiency, and robustness for safety-critical perception on resource-constrained edge devices. Through a principled hardware–algorithm co-design methodology, this work moves beyond the conventional sequential approach of designing a model and then compressing it, offering an integrated solution tailored for the extreme demands of environments such as construction sites.

The core contributions consist of three synergistic innovations. First, the Adaptive Feature Golden-Section Fusion (AFGS) module dynamically regulates cross-scale information flow via a learnable conflict gate, explicitly mitigating feature conflicts between objects of vastly different sizes. This innovation yielded a significant 9.3% improvement in Average Precision for challenging small-scale safety objects. Second, our Hardware-Conscious NAS (HC-NAS) co-optimizes model topology, structured sparsity, and quantization sensitivity within a unified search loop, guided by a multi-objective reward function that incorporates domain-specific risk priors. This automated process discovered the Pareto-optimal CSPDarknet-AFGS-INT8 configuration, which achieves a state-of-the-art performance of 89.4% mAP@0.5 at only 1.8 W on a Jetson TX2, representing a substantial leap in power efficiency. Third, and most critically, we introduced a risk-weighted evaluation paradigm and loss function grounded in OSHA fatality statistics. This strategic alignment of the model’s optimization goal with real-world safety consequences resulted in a 34% reduction in high-consequence false negatives for the most lethal hazards.

Comprehensive validation on our curated ConSafe benchmark and multiple public datasets confirms MMDet-Edge’s superiority over a wide range of state-of-the-art edge detectors, not only in overall accuracy but also in risk-aware metrics and cross-domain generalization. The framework’s robustness was further demonstrated through rigorous stress testing under adverse environmental conditions. The framework’s efficacy is further validated by long-term industrial deployment. Integrated into a holistic monitoring system across three large-scale, active construction sites, MMDet-Edge enabled real-time, reliable detection of five safety-critical classes, directly contributing to a measured 22% reduction in reportable safety incidents—a clear testament to its practical efficacy and impact.

Beyond its immediate application in construction safety, this work offers a broader methodological blueprint for certified edge intelligence. It demonstrates that through (i) architectural synergy co-optimizing neural topology and deployment parameters, (ii) principled alignment of technical metrics with domain-specific risk hierarchies, and (iii) a seamless pipeline from algorithmic design to containerized field deployment, AI systems can be engineered to be not only computationally intelligent but also inherently safe, reliable, and trustworthy. For future work, we plan to address remaining challenges such as performance under extreme specular reflections via multi-modal sensing and to extend the co-design paradigm to other safety-critical edge domains.

## Figures and Tables

**Table 1:** Overall Detection Accuracy Comparison.

Model	mAP@0.5 (%)	mAP@0.5:0.9 (%)	Helmet AP (%)	Fire AP (%)	Vest AP (%)	SIS (%)
YOLOv7-tiny [19]	83.5±0.4	62.1±0.5	84.8±0.6	83.1±0.7	89.7±0.4	0.60
YOLOv8n [9]	84.7±0.3	63.2±0.4	84.7±0.3	84.2±0.6	90.1±0.3	0.63
YOLOv11n [20]	85.5±0.3	64.0±0.4	87.0±0.5	85.0±0.5	90.5±0.3	0.66
NanoDet-Plus [21]	82.1±0.5	60.8±0.6	83.7±0.7	81.9±0.8	88.3±0.5	0.58
PP-PicoDet-S [22]	85.8±0.3	64.5±0.4	87.5±0.4	85.8±0.5	91.0±0.3	0.68
RT-DETR-l [23]	88.1±0.2	66.8±0.3	89.5±0.3	88.2±0.4	90.8±0.3	0.77
MMDet-Edge	89.4±0.2	67.9±0.3	91.2±0.2	90.1±0.3	88.9±0.4	0.83

**Table 2:** Cross-dataset generalization performance (Train: ConSafe).

Model	SHWD (Helmet) mAP@0.5 (%)	FireNet (Fire/Smoke) mAP@0.5 (%)	ConstDet (Mapped Classes) mAP@0.5 (%)
YOLOv8n	80.3	78.5	75.8
PP-PicoDet-S	83.5	81.2	79.4
MMDet-Edge	85.1	83.7	82.0

**Table 3:** Edge Deployment Performance on Jetson TX2.

Model	Throughput (FPS)	Power (W)	Peak Memory (GB)	Mean Latency (ms)	Latency σ (ms)
YOLOv7-tiny	27.3±2.5	2.83±0.18	1.79	36.6	18.3
YOLOv8n	22.1±3.1	3.24±0.21	2.11	45.2	24.7
NanoDet-Plus	29.8±1.9	2.15±0.15	1.42	33.5	12.1
PP-PicoDet-S	30.5±1.5	2.05±0.10	1.38	32.8	8.9
MMDet-Edge	31.2±0.7	1.82±0.05	1.21	32.1	6.4

**Table 4:** Cross-device deployment results (TensorRT INT8; batch = 1).

Device	Model	FPS	Mean Latency (ms)	Power (W)	Energy/Frame (J)	Peak Mem (GB)
Jetson TX2	MMDet-Edge	31.2	32.1	1.82	0.058	1.21
Xavler NX	MMDet-Edge	48.5	20.6	7.5	0.155	1.35
Orin Nano (8 GB)	MMDet-Edge	65.0	15.4	6.2	0.095	1.40

**Table 5:** Ablation Study of MMDet-Edge Components.

Configuration	mAP@0.5 (%)	Power (W)	SIS
Baseline (YOLOv8n)	84.7	3.24	0.63
+AFGS Module	87.8	3.18	0.72
+Hardware-Conscious NAS	89.4	1.82	0.79
+Risk-Weighted Loss (Full System)	89.4	1.82	0.80

**Table 6:** Neck module ablation (same backbone/head).

Model	mAP@0.5	Small-Object AP (Helmet)	Params (M)	FLOPs (G)	INT8 Latency (ms)
PAN/FPN	84.7	84.7	6.2	9.8	31.5
BiFPN [24]	86.1	86.5	7.1	11.5	35.2
ASFF [25]	86.5	87.0	7.8	12.3	37.8
CBAM-FPN	85.9	86.2	6.9	10.9	33.1
AFGS	87.8	89.2	7.5	11.8	34.5

**Table 7:** Risk-aware objective comparison (same AFGS architecture).

Loss/Training Objective	mAP@0.5	SIS-mAP	High-Risk FNR (Helmet)	High-Risk FNR (Fire)
Focal (baseline)	87.8	0.72	12.5%	15.2%
Class-balanced focal	87.5	0.75	11.8%	14.5%
LDAM-DRW	87.6	0.76	11.2%	14.0%
Inverse-frequency weighting	87.3	0.74	12.0%	14.8%
SIS-weighted (ours)	87.8	0.80	8.8%	12.1%

**Table 8:** Sensitivity analysis of NAS reward coefficients.

$\lambda_{1}$	$\lambda_{2}$	$\lambda_{3}$	Best mAP@0.5	Best Power (W)	Best FPS
0.10	0.10	0.05	89.5	2.10	28.5
0.17	0.23	0.11	89.4	1.82	31.2
0.25	0.35	0.15	88.7	1.65	35.0
0.35	0.50	0.20	87.5	1.45	38.5

**Table 9:** Environmental Robustness Analysis.

Condition	YOLOv8n (%)	YOLOv7-Tiny (%)	MMDet-Edge (%)	Δ Improvement
Baseline (Normal)	84.7	83.5	89.4	-
Heavy Rain (>50 mm/hr)	72.1	73.8	87.2	+10.4
Backlight (10,000 lux)	64.3	67.2	85.4	+16.4
Dust Occlusion (40%)	71.5	70.1	84.1	+7.9
Motion Blur (30 px)	68.7	69.8	82.3	+8.9
Low Light (5 lux)	66.2	65.7	80.9	+10.0

**Table 10:** Cross-site generalization.

Train Sites → Test Site	mAP@0.5	SIS-mAP	High-Risk FNR
A + B → C	85.2	0.78	10.5%
A + C → B	84.8	0.77	11.0%
B + C → A	86.0	0.79	9.8%

## Data Availability

Data are contained within the article.
